# THE NATURE OF PEER WORKPLACE INCIVILITY AND BULLYING AMONG RESIDENTIAL CARE AIDES IN LONG-TERM CARE

**DOI:** 10.1093/geroni/igz038.2569

**Published:** 2019-11-08

**Authors:** Heather A Cooke, Kaitlin Murray, Jennifer Baumbusch, Lisa Kelly

**Affiliations:** 1 University of British Columbia, Vancouver, British Columbia, Canada; 2 Simon Fraser University, Vancouver, British Columbia, Canada; 3 Sienna Senior Living, Vancouver, British Columbia, Canada

## Abstract

Residential care aides (RCAs; unregulated workers also known as certified nursing assistants or personal care assistants) provide much of the hands-on care in long-term residential care (LTRC). While many RCAs report being exposed to violent or aggressive acts from residents, we know little about their exposure to incivility and bullying from their colleagues. This is a significant knowledge gap as increased workplace incivility and bullying is associated with specific gender-dominated fields, hierarchical and stressful work environments, and low job autonomy, all of which characterize LTRC. Drawing on data from a critical ethnography examining workplace incivility and bullying among RCAs in a rural, not-for-profit care home, this presentation explores the types of incivility and bullying encountered by RCAs, and the contextual factors impacting their experiences with such behaviors. To date, more than 50 hours of participant observation, and 20 in-depth interviews with RCAs, licensed practical nurses, support staff, and management have been conducted. Findings illustrate the pervasiveness of incivility; while bullying acts (repeated, hostile behaviors intended to undermine, humiliate or injure) were rare, incivility (low-intensity acts with an ambiguous intent to harm) was an almost daily occurrence. Commonly-occurring behaviors included ignoring and refusing co-worker’s requests for help, social exclusion, acting impatient with, blaming and criticizing co-workers, and insisting on getting one’s own way. Chronic staffing shortages, staffing arrangements, and workload issues exacerbated RCAs’ experiences. Findings provide an important first step in understanding the nature of workplace incivility and bullying in LTRC.

